# PEDIATRIC ASTHMA: IMPACT OF THE DISEASE IN CHILDREN RECEIVING OUTPATIENT TREATMENT IN SOUTHERN BRAZIL

**DOI:** 10.1590/1984-0462/2020/38/2018398

**Published:** 2020-07-13

**Authors:** Cristian Roncada, Rodrigo Godinho de Souza, Daniela Duarte Costa, Paulo Márcio Pitrez

**Affiliations:** a Pontifícia Universidade Católica do Rio Grande do Sul, Porto Alegre, RS, Brazil.

**Keywords:** Quality of life, Asthma, Management, Children, Adolescents, Qualidade de vida, Asma, Manejo, Crianças, Adolescentes

## Abstract

**Objective:**

To evaluate the impact of pediatric asthma on patients of a specialized outpatient clinic in Southern Brazil.

**Methods:**

The study included children aged 8 to 17 years old with asthma diagnosis (mild, moderate and severe) under treatment at the asthma clinic of Hospital São Lucas da Pontifícia Universidade Católica do Rio Grande do Sul (PUCRS), Brazil. Measurements of spirometry, quality of life, disease control and atopy tests were applied.

**Results:**

A total of 66 children were included in the study and divided into groups, according to the severity of the disease: mild, moderate or severe asthma. The results showed similarities in both the treatment and the impact of asthma between groups, except for adherence to treatment: the group with mild asthma showed least adherence to treatment, and the group with severe asthma, greater adherence (p=0.011). As to school absenteeism, the group with severe asthma showed higher frequency (p=0.012), with over 10 days per year (p=0.043). Spirometry showed lower volume/capacity for the group with moderate asthma, followed by the groups with severe and mild asthma. All groups had a high prevalence of allergic asthma, with mites as the main allergens. For quality of life (QOL), and health-related quality of life (HRQOL) levels, there were no differences between groups. In addition, the values were close to the acceptable levels for the total score and for each one of the six domains. The same occurred for the HRQOL-asthma module.

**Conclusions:**

QOL and HRQOL present acceptable levels regardless of the severity of the disease.

## RESUMO

**Objetivo:**

Avaliar o impacto da asma pediátrica de pacientes em acompanhamento ambulatorial em um centro de referência em pneumopediatria do Sul do Brasil.

**Métodos:**

Participaram do estudo crianças com idade entre oito e 17 anos, com diagnóstico de asma (leve, moderada e grave), em acompanhamento no ambulatório de asma do Hospital São Lucas da Pontifícia Universidade Católica do Rio Grande do Sul (PUCRS). Foram verificadas medidas de espirometria, avaliação dos níveis de qualidade de vida, controle da doença e teste de atopia.

**Resultados:**

Sessenta e seis crianças participaram do estudo, divididas em três grupos (asma leve, moderada e grave). Evidenciaram-se semelhanças tanto no tratamento quanto no impacto da asma, exceto para a adesão ao tratamento (p=0,011), em que o grupo de asma leve é o que menos adere e o grupo de asma grave o que mais adere ao tratamento. Em relação ao absenteísmo escolar, o grupo de asma grave apresentou o maior valor (p=0,012), com mais de dez dias/ano (p=0,043). As espirometrias demonstram menor volume/capacidade para o grupo de asma moderada, seguido do grupo de asma grave e asma leve. Os grupos possuem alta prevalência de asma alérgica, tendo os ácaros como os principais alérgenos. Quanto à qualidade de vida (QV) e à qualidade de vida relacionada à saúde (QVRS), não houve diferença entre os grupos. Além disso, os valores apresentados estão próximos aos níveis aceitáveis, tanto para o escore total quanto para os seis domínios analisados. O mesmo ocorre para o módulo QVRS-asma.

**Conclusões:**

Os níveis de QV e de QVRS demonstram-se aceitáveis, independentemente da gravidade da doença.

## INTRODUCTION

Asthma is one of the most frequent chronic diseases in the pediatric population worldwide, considered a low-lethality disease, but with high rates of morbidity, which makes it a serious public health problem. Its affections are high to the point of being one of the main diseases in terms of emergency visits in emergency care units and hospital admissions.^1^ Its prevalence has been steadily increasing in the pediatric population, and despite advances in the management and treatment of the disease, high rates of morbidity and mortality are alarming.^1^ ^,^ ^2^

This respiratory disease is the result of three specific characteristics: airway obstruction, inflammation and bronchial hyperresponsiveness. Such characteristics cause three clinical manifestations: dry cough, dyspnea and wheezing. Knowing this, as well as its manifestations, the World Health Organization (WHO) developed a series of guidelines involving the treatment, self-management and control of asthma.^1^ During crises, the patient must be treated immediately with bronchodilator, enabling increased airflow.^2^ Self-management of asthma involves education and patient awareness of the importance of treatment and self-control when in crisis. Asthma control occurs with the practice of exercises, which strengthen the muscles involved in breathing, and adherence to the treatment prescribed by the pediatrician.^1^

Management for the treatment of children and adolescents with asthma is based on anamnesis, clinical examination and, whenever possible, pulmonary function tests (spirometry) and assessment of allergies.^2^ An important factor in the classification of disease severity is the assessment of quality of life (QOL), which is an individual perception, of multiple factors that directly or indirectly affect life, for example, physical, cultural, social, environmental and emotional aspects.^3^ When QOL is affected due to a specific disease, this is called health-related quality of life (HRQOL),^3^ which is measured with the aid of specific questionnaires, such as the Kinder Lebensqualität Fragebogen (KINDL-R),^4^ the Pediatric Asthma Quality of Life Questionnaire (PAQLQ)^5^, and the Pediatric Quality of Life Inventory TM (PedsQL TM).^6^ Another important factor is disease control, which, likewise, is assessed using specific questionnaires; the most used ones are the Asthma Control Test (ACT,^7^ the Asthma Control Questionnaire (ACQ)^8^, and the Global Initiative for Asthma (GINA).^1^ With the application of all the methods described, diagnosis, treatment, self-management, control, physical activity and periodic evaluation, the QOL of patients becomes much better and easier to maintain at acceptable levels. Based on these facts, the objective of the study was to evaluate the impact of pediatric asthma in patients undergoing outpatient follow-up at a referral center for pneumopediatrics in Southern Brazil.

## METHOD

From March to December 2014, a cross-sectional study was carried out in children with a clinical diagnosis of persistent asthma (mild, moderate and severe), based on the GINA guidelines,^1^ undergoing outpatient follow-up at a pediatric asthma reference center in Porto Alegre, Rio Grande do Sul. The sample was selected by convenience criterion, with the participation of children aged between eight and 17 years old in follow-up for at least six months, with no history of physical or cognitive disabilities that could compromise the assessments of outcome.

At the time of inclusion in the study, a clinical questionnaire was applied, containing questions with the characterization of the sample, the history of crises and treatments for the disease. In addition, pulmonary function parameters/indexes - spirometry were assessed: forced expiratory volume in one second (FEV_1_), forced vital capacity (FVC), ratio of FEV_1_ and FVC (FEV_1_/FVC) and forced expiratory flow between the 25 and 75% percentiles (FEF25-75%), presented by Z score-both basal and after the use of bronchodilator (400 µg of salbutamol);^9^ of QOL, with the KINDL-R questionnaire,^10^ composed of 24 generic items about physical and emotional well-being, self-esteem, family, friends and school and 15 items related to health (asthma). Disease control was also assessed with the ACT,^11^ with acceptable levels with scores equal to or greater than 20 points, adherence to treatment and perception of changes in health (containing a question for each item), body mass index-BMI (weight/height^2^), presented by Z score,^12^ and physical activities,^13^ consisting of items about the practice of physical activity and the time spent on them in the last seven days, as well as items related to the time spent in front of screens (television, video game and computer).

For the purpose of assessing atopy, the skin prick test was applied,^14^ with immediate reading on patients’ forearms, containing eight types of antigen for evaluation in asthma (*Dermatophagoides pteronyssinus*, *Dermatophagoides farinae*, *Blomia tropicalis*, grasses, cockroach, air fungi, dog epithelium and cat epithelium), in addition to positive (histamine) and negative (serum) testing. Initially, tests were considered valid by presenting a papule≥3 mm for histamine (positive test) and not presenting a papule for serum (negative test) after 15 minutes of application. After this check, patients with a papule≥3 mm were considered positive (atopic) for any of the eight antigens tested. All tests applied in the present study were carried out by a team previously trained and qualified for such measures and assessments, with questionnaires administered in the form of interviews and objective tests, according to the rules stipulated by the collection instruments previously mentioned.

For the purposes of statistical analysis, categorical values are expressed by absolute and relative numbers (N%), and continuous values by means and standard deviations. For comparison between groups, chi-square tests and analysis of variance (ANOVA) are used, according to the analyzed variable. In addition, for the configuration of the study and comparison between groups, the minimum acceptable sample size was 19 subjects per group (mild, moderate and severe), with an effect size (p) of 0.3 point, confidence level (β) minimum of 80% and sampling error (α) of up to 5%.

The study was approved by the Research Ethics Committee (CEP) of Pontifícia Universidade Católica do Rio Grande do Sul (PUCRS), under substantiated opinion No. 1.535/2011. Both patients and guardians consented to participate in the study by the consent term (children/adolescents) or the free and informed consent (guardians).

## RESULTS

The sample of this study was composed of 66 children with an average age of 10.5±2.1 years, 40 (61%) of whom were male; 43 (65%) belonging to the economic and social class C; 51 (77%) of Caucasian ethnicity and severity of mild, moderate and severe asthma (32, 36 and 32%, respectively); 33 (50%) with BMI above normal; 30 (45%) with acceptable levels of asthma control; 32 (48%) with acceptable levels of physical activity (active); and 53 (80%) with high rates of physical inactivity, with time in front of screens (TIFS) - televisions, video games or computers - ≥2 hours/day. In addition, mothers are the main caregivers (companions) in medical consultations (51; 77%). Table 1 shows sample characterization values by asthma groups (mild - n=21, moderate - n=24, and severe - n=21).

**Table 1:** Characterization of the groups with asthma (mild, moderate and severe).

Categorical variables	Mild		Moderate		Severe		p-value
	n=21	%	n=24	%	n=21	%	
Accompanying person (mother)	15	71.4	20	83.3	16	76.2	0.560
Economic and social class (class C)	15	71.4	14	58.3	14	66.7	0.649
Gender (male)	17	80.9	14	58.3	9	42.9	0.061
Ethnicity (caucasian)	18	85.7	19	79.2	14	66.7	0.404
BMI (Z score)							
Eutrophic	9	42.9	12	50.0	12	57.1	0.676
Overweight	5	23.8	6	25.0	4	19.0	
Obesity	7	33.3	6	25.0	5	23.8	
Physical activity level (active)	10	47.6	13	54.2	9	42.9	0.751
Physical inactivity level (≥2 hours/day)*	18	85.7	20	83.3	15	71.4	0.461
Asthma control test (controlled)	10	47.6	11	45.8	9	42.9	0.953
Continuous variables	AV	±SD	AV	±SD	AV	±SD	p-value
Age	11.0	±2.6	10.8	±2.3	9.7	±1.6	0.106
First asthma attack (in months)	28.7	±7.6	34.0	±9.0	20.4	±5.3	0.362
Outpatient care (in months)	39.5	±9.6	55.4	±5.5	35.3	±4.9	0.294
Asthma control test (ACT)	18.9	±3.5	18.3	±4.0	18.0	±4.0	0.740
Physical activity time (in minutes)	320.0	±41.3	224.2	±22.3	196.9	±22.7	0.413
Physical downtime (in hours)	3.6	±2.1	3.9	±2.8	2.9	±1.7	0.332

n: number of participants; %: percentage of participants; BMI: body mass index; AV: average; SD: standard deviation; *tendency to physical inactivity for staying in front of screens (televisions, computers or video games) for more than 2 hours/day.

Table 2 shows the categorical values, as well as the comparisons between the three asthma groups, demonstrating that there are similarities in both treatment and asthma impact, with significant differences only for adherence to treatment (p=0.011): the group with mild asthma is the least adherent to the treatment, and the group with severe asthma is the one that adheres the most. In addition to this outcome, differences were found for school absenteeism: the group with severe asthma was the one with the highest scores (p=0.012), with the longest periods of absence from school (more than ten days/year; p=0.043).

**Table 2:** Comparison between treatment and asthma impact among asthma groups (mild, moderate and severe).

	Mild		Moderate		Severe		p-value
	n=21	%	n=24	%	n=21	%	
Asthma treatment							
Crisis prescription	17	80.95	18	75.00	13	65.00	0.508
Continuous treatment for asthma	14	66.67	19	79.17	19	90.48	0.173
Forget to administer treatment							
Never	0	0.00	3	15.79	9	47.37	0.011*
Sometimes	14	100.00	15	78.95	10	52.63	
Ever	0	0.00	1	5.26	0	0.00	
Change in health after treatment							
Better	10	71.43	14	73.68	13	68.42	0.374
Equal	2	14.29	3	15.79	3	15.79	
Worse	2	14.29	2	10.53	3	15.79	
Impact of asthma over the last 12 months							
Dry cough at night	14	66.67	19	79.17	19	90.48	0.143
Sleep disturbed by asthma	14	66.67	16	66.67	18	85.71	0.965
Exercise-induced asthma	14	66.67	15	62.50	15	71.43	0.820
Asthma attacks	20	95.24	21	87.50	19	90.48	0.652
1 to 3 times	13	61.90	10	41.67	4	19.05	0.169
4 to 7 times	2	9.52	1	4.17	4	19.05	
8 to 11 times	1	4.76	4	16.67	3	14.29	
At least one crisis a month	4	19.05	6	25.00	8	38.10	
Medical consultation for asthma crisis	20	95.24	21	87.50	19	90.48	0.652
Did not consult (treated at home)	17	85.00	14	66.67	10	52.63	0.082
In primary care	3	15.00	5	23.81	7	36.84	
In tertiary care	0	0.00	2	9.52	2	10.53	
Hospitalization	0	0.00	1	7.14	2	13.33	0.379
School absenteeism	10	47.62	16	66.67	19	90.48	0.012*
1 to 5 days	6	60.00	5	31.25	4	21.05	0.043*
6 to 10 days	2	20.00	6	37.50	5	26.32	
More than 10 days	2	20.00	5	31.25	10	52.63	

n: number of participants; %: percentage of participants; *significance value with p<0.05, chi-square test being applied.

Table 3 shows the continuous values (Z score) for representing pulmonary function (spirometry), comparing the three groups of asthma, showing a difference between basal values (FEV_1_, FVC and FEF25-75% - p=0.002, p=0.001 and p=0.036, respectively) and post-use of bronchodilator (FEV_1_ and FVC - p=0.008 and p=0.037, respectively). The values show lower volume/capacity for the group with moderate asthma, followed by the group with severe asthma and the group with mild asthma. In addition, when comparing the difference between pre- and post-bronchodilator, no statistical differences were found.

**Table 3:** Comparison of lung function (spirometry) among asthma groups (mild, moderate and severe).

	Mild		Moderate		Severe		p-value
	AV	±SD	AV	±SD	AV	±SD	
Pre-bronchodilator (Z score)							
FEV_1_	0.89	±1.32	-0.67	±1.48	0.61	±1.77	0.002*
FVC	1.49	±1.25	-0.18	±1.46	0.85	1±.66	0.001*
FEV_1_/FVC	-0.79	±1.04	-0.77	±1.25	-0.42	±1.29	0.522
FEF_25-75%_	-0.30	±1.17	-1.00	±1.28	0.10	±1.76	0.036*
Post-bronchodilator (Z score)							
FEV_1_	1.69	±1.48	0.24	±1.63	1.28	±1.60	0.008*
FVC	1.81	±1.60	0.41	±2.12	1.21	±1.51	0.037*
FEV_1_/FVC	-0.21	±0.67	-0.14	±1.14	0.00	±1.07	0.778
FEF_2575%_	0.53	±0.92	0.13	±1.64	0.71	±1.52	0.371
Pre/post-bronchodilator difference (%)							
FEV_1_	8.27	±8.86	10.51	±9.73	7.60	±7.96	0.519
FVC	3.19	±5.19	6.31	±9.80	4.37	±5.69	0.359
FEV_1_/FVC	13.51	±22.45	15.02	±26.08	10.00	±23.03	0.777
FEF_25-75%_	23.32	±23.66	22.34	±23.88	13.18	±18.46	0.267

AV: average; SD: standard deviation; *significance value with p<0.05, independent Student t-test being applied; FEV_1_: forced expiratory volume in one second; FVC: forced vital capacity; FEV_1_/FVC: ratio between forced expiratory volume in one second and forced vital capacity (Tiffenau); FEF_25-75%_: forced expiratory flow in the 25 and 75% percentiles.

**Figure 1:**
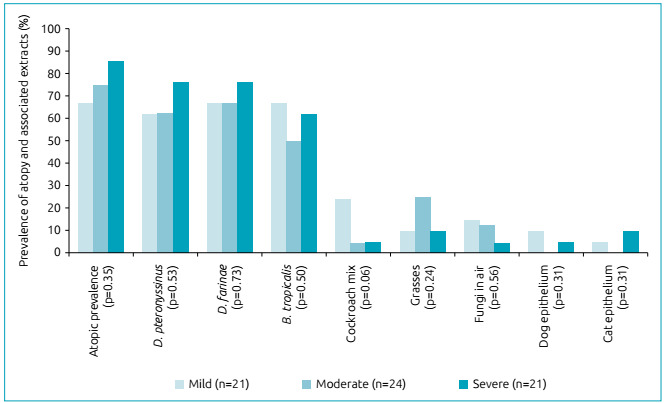
Assessment of atopic prevalence by asthma severity groups.

Figure 1 represents the prevalence levels of atopy, with no differences between the groups, but, at the same time, demonstrating that they have a high prevalence of allergic asthma, with mites being the main factors of atopy and domestic animals (dogs and cats) those with the lowest prevalence rates.

**Figure 2:**
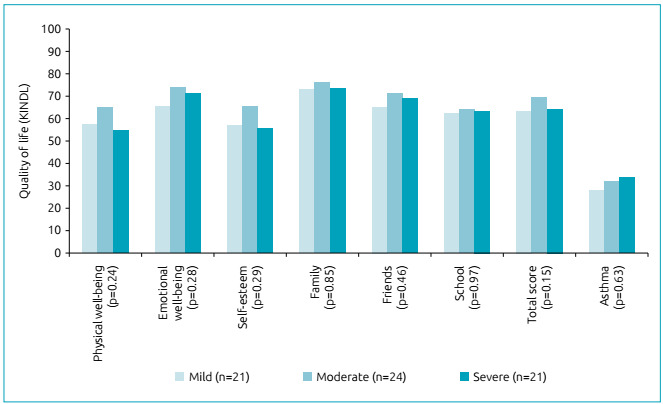
Assessment of quality of life by asthma severity groups.

Figure 2 represents QOL levels (generic), as well as HRQOL-asthma levels, with no differences between groups. In addition, the values presented are close to acceptable levels (≥ 70 points) both for the total score and for the six domains composed of the generic questionnaire. As for the HRQOL-asthma module, in which the acceptable values are inversely proportional, the values were also close to the acceptable levels (≤30 points).

## DISCUSSION

The results show that the impact of pediatric asthma is high, regardless of the severity of the disease, compromising aspects of daily life due to low adherence to treatment, making it difficult to control, increasing levels of disease recurrence (daytime, nighttime and exercise-induced symptoms), emergency visits and hospitalizations. The results also point to an increase in school absenteeism, BMI, physical inactivity and atopy. In addition, there are differences in lung function, especially for patients with moderate asthma (decreased capacity/volume). However, the QOL and HRQOL-asthma levels are acceptable, regardless of the severity of the disease.

In assessing BMI, the results demonstrate that children with a clinical diagnosis of asthma have high rates of overweight and obesity, regardless of the severity of the disease. In a study with 324 urban asthmatic children, Holderness et al.^15^ reported that patients have an overweight rate of 15% and an obesity rate of 31%, identifying that children with limited physical activity have a significantly higher chance of being overweight or obese-2.1 *Odds Ratio* (OR), 95% confidence interval (95% CI) 2~3.8. In addition, children with symptoms of poorly controlled asthma, compared to children with milder symptoms, report having limitations to perform physical activities (58 *versus* 43%, p=0.01). The authors conclude that urban children with persistent asthma have high rates of overweight and obesity, which generates limitations to physical activities, resulting in uncontrolled disease in 47% of cases. In another study, Chen et al.^16^ warn of the risk of increased adiposity in the short term, which may increase the incidence or recurrence of symptoms of childhood asthma, besides increased airway inflammation.

In the present study, it was demonstrated that 48% of the evaluated children have acceptable levels of physical activity (active), and the others (52%), high levels of physical inactivity, with the aggravating fact that 80% of these have high scores for TIFS (≥2 hours daily). Studies^17^ ^,^ ^18^ ^,^ ^19^ point out that the levels of physical inactivity have been gradually increasing in children and adolescents, simultaneously, which corroborates the importance of evaluating possible risks associated with physical inactivity, such as irregular eating habits or TIFS≥2 hours daily. Lochte et al.,^20^ in their meta-analysis, assessed the relation between asthma symptoms and physical inactivity, pointing out evidence that children and adolescents with low levels of physical activity are at increased risk for the appearance of new asthma symptoms (disease recurrence).

Furthermore, Hoare et al.,^18^ in a systematic review, assessed the association between sedentary behavior and mental health problems in children and adolescents, pointing out strong evidence for the positive relation between depressive symptoms and TIFS, especially for children and adolescents with a mean TIFS between two and three hours daily. Moderate evidence was also found for the relation between self-esteem and sedentary behavior, indicating lower levels of self-esteem among those who reported higher levels of TIFS (watching TV and using the computer). Both studies indicate that such findings serve as an alert for the importance of assessing the negative impact on pediatric health related to lifestyle changes, attributed to the new behavioral, environmental and social trends of this population.

Regarding the assessment of asthma control indexes, the results of the present study demonstrate that 55% of the assessed population does not have acceptable total score scores for disease control - ACT questionnaire score≥20 points - regardless of the severity of the disease. Halwani et al.^21^ evaluated 297 children and pointed out that most patients (60.3%) had uncontrolled symptoms, of which intermittent asthmatics had better scores on the total ACT score compared to patients with more severe symptoms, which was attributed to the lack of adherence to treatment or the inappropriate use of inhalation devices. In another study, Saito et al.^22^ evaluated 229 asthmatic patients and reported that the management for asthma control must take into account the biological, psychological and treatment adherence problems, thus leading to a more proactive and aimed at better disease control.

As to the treatment for asthma, as well as its adherence, the present study shows that 74% of asthmatics have a crisis prescription for preventive treatment, and 79% use continuous medication, regardless of the severity of the disease. About half of patients with severe asthma, 15% of patients with moderate asthma and no patient with mild asthma report never forgetting to administer the medication. Similar findings were noted in a Dutch cohort,^23^ in which children with a history of high treatment adherence had higher rates of exacerbations during follow-up compared to children with lower treatment adherence rates. In conclusion, the study points out that the characteristics of children with good adherence are compatible with more severe asthma, suggesting that adherence is driven by the need for treatment or the intensity of medical monitoring.

Regarding emergency care, patients with severe asthma were those with the highest prevalence (47%) and the highest frequency of hospitalization (13%). Such findings demonstrate that the lower the severity of the disease, the lower the impact rates of asthma, with residential rescue treatment, reducing the chances of hospitalization, in addition to finding significant differences regarding school absenteeism: children with mild asthma miss school days for shorter periods and children with severe asthma, for longer periods (p=0.043). In a cross-sectional study carried out with 715 asthmatics, Roncada et al.^24^ demonstrated that asthma morbidity is high in this population (68%), with reports of recurrent symptoms of this disease over the last 12 months. Among the 715 students, 56% attended at least one medical appointment for asthma, and only 24% underwent specialized medical follow-up, with half of the children using oral corticosteroids in the last 12 months and 8% being hospitalized for the disease, with a prevalence of school absenteeism of 57%.

In assessing lung function (spirometry), comparing it to asthma severity, the results of the present study demonstrate a difference between basal values for FEV_1_, FVC and FEF25-75% (p=0.002, p=0.001 and p=0.036, respectively) and post-use of bronchodilator for FEV_1_ and FVC (p=0.008 and p=0.037, respectively), with worse lung volume/capacity for the group with moderate asthma group, followed by the group with severe asthma, with the best scores belonging to the group with mild asthma. These findings may be related to the treatment of groups and the time of year evaluated, considering that, in the referral center, patients with mild and moderate asthma have drug reduction in the periods of hot season (summer), with the group with severe asthma continuing to have severe symptoms 12 months a year. However, when comparing the difference between pre- and post-bronchodilator, no differences were found with the use of 400 µg of salbutamol. A possible answer to these findings is the fact that patients in the group with mild asthma have better lung function, as described in the literature,^25^ and those in the group with severe asthma are prescribed treatment with Omalizumab,^26^ making the group with moderate asthma the one with the worst lung function scores. In addition, a recently published study^27^ demonstrates that the bronchodilator response, correlating with asthma control and assessed by the ACT questionnaire, did not show significant differences before and after the administration of 400 µg of salbutamol between the groups.

In assessing atopic hypersensitivity levels using the skin prick test, even though there was an increase in prevalence according to severity, the differences were not significantly relevant (mild asthma=67%, moderate asthma=75%, and severe asthma=86% ; p=0.352). Nonetheless, even though no differences were found between the groups, atopic prevalence was relatively high compared to another study in the same region,^28^ which showed minimum values of 67% for the group with mild asthma. Household dust mites were mainly responsible for the high diagnosis, with a positive response in 76% of cases of severe asthma for *Dermatophagoides pteronyssinus* and *Dermatophagoides farinae*, followed by 67% for cases of mild asthma for *Blomia tropicalis*. The epitheliums of dogs and cats had the lowest rates of atopic prevalence. Similar findings for the prevalence of atopy are presented in a study conducted in the same region, in which 85% of children were atopic, with *Dermatophagoides farinae*, *Dermatophagoides pteronyssinus* and *Blomia tropicalis* as their main triggering factors.^29^

One of the main outcome assessments for the management of asthma is that of HRQOL, represented in this study as QOL (generic module) and HRQOL-asthma. Both assessments were found to be very close to normal standards, regardless of asthma severity. One of the possible answers to these findings is that children are being followed up on an outpatient basis, increasing the perception of HRQOL. Moreover, Matsunaga et al.^30^ demonstrated that QOL is directly related to the level of asthma control and severity, because children and adolescents with better control and lesser disease severity had better QOL. Thus, levels of asthma control and severity can influence the QOL of asthmatic patients and their families. These findings underscore the importance of adequate monitoring of this population, with an emphasis on factors that lead to an unfavorable outcome, such as lack of adherence to treatment, contact with triggering factors, inappropriate use of inhalation devices and inaccessibility to medicines and medical services. Perhaps, these last two topics are the reasons for the findings of this study not having demonstrated any differences in QOL scores between the groups with mild, moderate and severe asthma.

The assessment of adherence to treatment based on the report of the person responsible for the patient is one of the study limitations. In some cases, such factor could under or overestimate the data. Hence, the ideal practice would be using a metered-dose inhaler that recorded the date and time of use of the medication, a resource which was not available.

Although the clinical characteristics of children with mild, moderate and severe asthma are similar in the present study, the mechanisms and risk factors should be better studied, as well as their association with the recurrence of symptoms, lifestyle, adherence to treatment, lung function, atopy and QOL levels. In this way, we can increase the levels of asthma control, reducing the global burden of the disease and providing better HRQOL for these children, adolescents and their family members.
